# A Cost-Effectiveness Analysis of India’s 2008 Prohibition of Smoking in Public Places in Gujarat

**DOI:** 10.3390/ijerph8051271

**Published:** 2011-04-26

**Authors:** Elisabeth A Donaldson, Hugh R Waters, Monika Arora, Beena Varghese, Paresh Dave, Bhavesh Modi

**Affiliations:** 1 Institute for Global Tobacco Control, Johns Hopkins Bloomberg School of Public Health, 627 N. Washington Street, 2nd Floor, Baltimore, MD 21205, USA; 2 Rand Corporation, 1776 Main Street, Room 4369, Santa Monica, CA 90401, USA; E-Mail: waters@rand.org; 3 Public Health Foundation of India, Second Floor, 4/2, Sirifort Institutional Area, August Kranti Marg, New Delhi, 110016, India; E-Mails: monika.arora@phfi.org (M.A.); beena.varghese@phfi.org (B.V.); 4 State Tobacco Control Cell, Department of Health & Family Welfare, Government of Gujarat, 3rd Floor, Block No. 5/2, Dr. Jivraj Mehta Bhavan, Sector No. 10, Gandhinagar-Gujarat 382010, India; E-Mails: pvdave1@rediffmail.com (P.D.); bhavmod@yahoo.com (B.M.)

**Keywords:** cost-effectiveness, public smoking bans, smoke free public places, secondhand smoke, tobacco smoking

## Abstract

Tobacco smoking and exposure to secondhand tobacco smoke are associated with disability and premature mortality in low and middle-income countries. The aim of this study was to assess the cost-effectiveness of implementing India’s *Prohibition of Smoking in Public Places Rules* in the state of Gujarat, compared to implementation of a complete smoking ban. Using standard cost-effectiveness analysis methods, the cost of implementing the alternatives was evaluated against the years of life saved and cases of acute myocardial infarction averted by reductions in smoking prevalence and secondhand smoke exposure. After one year, it is estimated that a complete smoking ban in Gujarat would avert 17,000 additional heart attacks and gain 438,000 life years (LY). A complete ban is highly cost-effective when key variables including legislation effectiveness were varied in the sensitivity analyses. Without including medical treatment costs averted, the cost-effectiveness ratio ranges from $2 to $112 per LY gained and $37 to $386 per acute myocardial infarction averted. Implementing a complete smoking ban would be a cost saving alternative to the current partial legislation in terms of reducing tobacco-attributable disease in Gujarat.

## 1. Introduction

Tobacco use constitutes a global epidemic that results in five million deaths each year [1]. If current trends in tobacco use continue, the number of tobacco-related deaths is expected to rise to eight million deaths annually by 2030—with 80 percent of these deaths ocurring in low- and middle-income countries (LMICs) [2].

Currently, about 10 percent of the world’s smokers live in India [1]. The 2009–2010 Global Adult Tobacco Survey, a nationally representative household survey, found that 34.6% of adults over the age of 15 in India currently use tobacco [3]. The prevalence of tobacco smoking in Gujarat, India, including those using smokeless and smoked tobacco concurrently is estimated to be 19.8% among males and 1.5% among females [3]. Most smokers in India consume bidis, small cigarettes containing, on average, 25 percent less tobacco than the average manufactured cigarette [4]. Despite the smaller amount of tobacco in bidis, they can produce more nicotine, carbon monoxide, and tar than the average manufactured cigarette due to the way users puff on them [5]. One nationally representative case-control study found that about 70% of smoking-related deaths in India take place during productive years of life between 30–69 years of age [4]. In addition, the study projected that starting in 2010; smoking will kill one million people each year [4].

Since 2005, the World Health Organization (WHO)’s Framework Convention on Tobacco Control (FCTC) offers a legally binding treaty that mandates evidence-based tobacco control policies and programs to reduce morbidity and mortality from tobacco use. Countries such as India that have ratified the FCTC are legally required to implement its provisions, including interventions to reduce secondhand smoke (SHS) exposure [6]. Article 8 of the FCTC requires signatories to adopt and implement measures to protect individuals from secondhand tobacco smoke (SHS) exposure in indoor work places, public transportation, and other public spaces.

Since ratifying the FCTC, India’s tobacco control policies and programs have undergone improvements to meet FCTC requirements. The central government of India enacted the *Cigarettes and Other Tobacco Products Act (COTPA)* on May 1, 2004 [7,8]. This legislation contains most of the areas covered by the FCTC articles—including a ban on smoking in public places. COTPA’s Section 4 describes the ban in public places and contains an exemption that allows separate smoking areas in restaurants with seating capacity for 30 or more persons, hotels with 30 or more rooms, and airports [7,8]. This is inconsistent with the FCTC’s Article 8, which recommends that all public places should be smoke free and should not allow designated smoking areas [6].

In an effort to strengthen tobacco control programs and legislation, the National Tobacco Control Programme (NTCP) was launched in 2007 in 18 districts across nine states in India, including two districts in Gujarat [7,8]. The central government provided resources through the NTCP to develop state- and district-level tobacco control cells. The tobacco control cells were established to implement tobacco control policies and programs. Activities, such as training and media campaigns, were conducted to assist implementation and enforcement of tobacco control laws, including COTPA’s Section 4.

In 2008, the central government’s Ministry of Health and Family Welfare proposed the *Prohibition of Smoking in Public Places Rules* to strengthen the existing COTPA legislation [7–9]. The Rules expand the smoking ban in COTPA’s Section 4 to include public spaces that were excluded in the original legislation; and the Rules define terms such as smoking and non-smoking areas [9]. In addition, the Rules provide instructions for enforcing the legislation, including details regarding the display of signs and the identification of focal points for implementing the law, such as school principals, airport managers, and others [7,9].

Although the Rules came into effect on October 2, 2008, compliance with the smoking ban remains low. Based on data from the 2009–2010 Global Adult Tobacco Survey, 32.8% of respondents reported exposure while visiting a public place during the past 30 days in the state of Gujarat, illustrating that exposure to secondhand smoke in public places remains high [3]. Approximately 45% of adults in Gujarat had visited a public place in the past 30 days that had a designated smoking area and 12% had observed smoking in a designated non-smoking area [3]. A monitoring study conducted in the city of Ahmadabad in Gujarat in early 2010 measured the level of air nicotine and particulate matter in public places and found detectable levels of secondhand smoke in all public places, including those where smoking is banned by the current legislation [10]. The highest concentrations of air nicotine were observed in restaurants, hookah bars, and entertainment venues with a designated smoking area [10].

Internationally, countries such as Ireland, New Zealand, England, as well as states and cities throughout the world, have enacted smoking bans in all indoor public places [11–13]. While long term compliance has been observed in the state of California in the US [14], implementation, or the broad application of partial and complete smoking bans, remains a challenge in many countries [15–18]. Therefore, the challenges that Gujarat faces are not unique when implementing a partial smoking ban. In comparison to the recommended per capita funding levels for state tobacco control programs in the US, the state of Gujarat does not have the recommended level of funding to effectively implement and enforce its current partial smoking ban [19]. Therefore, this analysis is one of the first to explore the funding for implementation and enforcement of the current smoking ban in a state in India and to explore whether the gains in health outcomes and savings in medical treatment costs can offset the total cost of implementing a complete smoking ban.

Currently, cost-effectiveness analyses of tobacco control policies and programs are limited, especially for low- and middle-income countries. Specifically, there are few, if any, studies published in the literature examining the cost-effectiveness of smoking bans in a national or sub-national setting. In the past several years, low- and middle-income countries such as India have seen an increased number of smoke free policies [20]. However, some of these policies do not meet the FCTC’s recommendations or are poorly implemented at the sub-national level [20]. Therefore, it is important to examine the cost-effectiveness of current smoke free policies to provide decision makers with the evidence needed to strengthen existing policies to meet FCTC requirements and to allocate additional funds for enforcement. Given the exceptions for smoking areas in India’s current legislation and the high levels of exposure to secondhand smoke reflected in recent data on compliance, there is a particular need for transparent cost-effectiveness analysis of smoke free legislation in India [3,20].

## 2. Methods

### 2.1. Model Overview

This study estimates the cost-effectiveness of implementing India’s *Prohibition of Smoking in Public Places Rules,* a partial smoking ban, in the state of Gujarat compared to implementation of a complete smoking ban that requires all public places to be smoke free [7]. The cost-effectiveness ratio is defined as the change in cost of the policy alternatives over the change in effectiveness relative to having a complete or partial smoking ban. Effectiveness is defined as the number of heart attacks or acute myocardial infarction (AMI) cases averted, and life years saved. We have taken the societal perspective in this cost-effectiveness analysis; the costs of both the intervention and the outcomes accrue to government, businesses, and individuals. The costs of conditions related to smoking and exposure to SHS were calculated using healthcare and consumer expenditure data from the 2004 National Sample Survey (NSS) in India [7]. This study uses standard methods of cost-effectiveness analysis to evaluate the cost of implementing the alternatives for one year against the years of life saved and AMI cases averted by reductions in smoking prevalence and secondhand smoke exposure over a ten-year analytic horizon [21].

### 2.2. Parameter Estimates

Parameter estimates used in the model including probabilities, costs, and outcome measures are summarized in Table 1 and below.

#### 

##### Prevalence of Tobacco Smoking

The prevalence of tobacco smoking in Gujarat, including bidis and cigarettes, as well as individuals using both smokeless and smoking tobacco is 19.8% among males and 1.5% among females according to the 2009–2010 Global Adult Tobacco Survey [3].

##### Effectiveness of Complete Smoking Ban

There are no studies in the published literature that evaluate the effectiveness of a complete smoking ban in India or in other lower middle-income countries. Studies conducted in the United States, Ireland, Norway, and Scotland have shown significant reductions in secondhand smoke exposure after a complete smoking ban based on measurement of environmental and biological markers, as well as self-reported exposure [26,30,31,41,42]. A study in Uruguay, the first upper middle-income country to implement a complete indoor smoking ban, found an overall reduction in air nicotine concentration of 91% one year after the ban [32].

Based on the available evidence, we estimated that legislation creating 100% smoke free public places would reduce exposure to secondhand smoke by 86% and current smoking prevalence by 3.4%. The complete smoking ban’s effectiveness on reducing SHS exposure was based on fine particulate matter (PM_2.5_) measurements before and two months after the implementation of Scotland’s smoking ban to provide a conservative estimate of the law’s impact [30]. Based on a recent literature review, the studies measuring active smoking through self-report and biological verification did not provide consistent evidence regarding changes in current smoking prevalence as a result of a complete smoking ban [43]. However, the twenty-three studies included in the review illustrated a trend toward a reduction in smoking prevalence [43]. Therefore, for the current study, the complete smoking ban’s impact on smoking prevalence was based on a cross-sectional survey conducted before and one year after Ireland’s complete smoking ban went into effect [25]. The survey identified active smoking prevalence in Ireland through self-report and cotinine concentrations among bar workers as compared to a cross-sectional survey of self-reported use among the general population [25]. Based on the available evidence, the impact of Ireland’s complete smoking ban on current smoking prevalence provides a conservative estimate of the anticipated reduction in adult smoking prevalence in Gujarat [25].

##### Effectiveness of 2008 Rules (Partial Smoking Ban)

Very few studies have assessed the effectiveness of a partial smoking ban. The impact of a partial ban on reducing exposure to secondhand smoke (SHS) was measured in Spain, The Netherlands, and several states in the US. A cross-sectional, population-based survey before and one year after Spain’s partial smoking ban went into effect observed a 22% reduction in self-reported SHS exposure [27]. The findings of observational studies in the US before and after several partial state smoking bans, observed a 21% reduction in SHS exposure [28], whereas a recent cross-sectional survey of self-reported exposure among restaurant and bar employees in the Netherlands found a 27% reduction [29].

A cross-sectional survey of representative national data explored the current smoking prevalence after a partial ban in South Korea and estimated a 1.9% reduction [23]. Levy *et al.* modeled the impact of a partial ban and determined that it would have approximately one-third the impact of a complete ban [24]. Based on the available evidence, we estimated that the 2008 Rules in Gujarat, a partial ban, would reduce exposure to secondhand smoke by 22% and the ban would have little or no change on adult smoking prevalence.

### 2.3. Costs

Both the costs of implementing a complete or partial ban on smoking in public places, as well as the direct medical costs associated with smoking-related disease were considered. All costs were converted into 2008 Rupees, corresponding to the year, October 2008, in which the Rules were implemented. Costs and benefits projected to occur more than one year in the future were discounted at 3%. A summary of costs is provided in Table 1 and below.

#### 

##### Cost of Tobacco-Attributable Disease in Adults

We used the Population Attributable Risk (PAR) to calculate the percentage of the cases of each smoking-related disease that can be causally linked to smoking and to exposure to secondhand smoke (SHS). PAR is the proportion of cases of a disease and associated mortality in a given population that can be considered to be causally related to exposure to a risk factor. PAR is calculated as:

$$\frac{\left(\text{Incidence}\;\text{in}\;\text{total}\;\text{population})-(\text{Incidence}\;\text{in}\;\text{unexposed}\;\text{group}\right)}{\left(\text{Incidence}\;\text{in}\;\text{total}\;\text{population}\right)}$$

The PAR can also be calculated using the Relative Risk (RR) for each condition, combined with prevalence data for the disease in question, as follows [44]:

$$\frac{\text{Prevalence}\times (RR-1)}{\text{Prevalence}\times (RR-1)+1}$$

Using prevalence estimates for health conditions related to smoking and exposure to SHS, we calculated the PAR for each of these conditions, using relative risks published in the literature [45,46]. The results show the number of episodes of each condition that can be attributed to smoking and exposure to SHS in Gujarat. Based on the number of episodes of each condition, tobacco-attributable disease costs for adults in Gujarat were calculated using national expenditure data [40].

Annual household and healthcare provider costs for each episode of health conditions related to smoking and exposure to SHS were calculated using healthcare and consumer expenditure data from the 2004 National Sample Survey (NSS) in India [40]. The 2004 NSS data was used to estimate the annual expenditure on smoking-related disease treatment, including visits, drugs, informal care giving, and travel costs [40,47].

##### Cost of Complete Smoking Ban

The cost of implementing and enforcing a complete smoking ban for a one-year period was estimated based on the programmatic costs developed by the WHO-CHOICE project [39]. The estimate included the cost of implementing non-price tobacco control interventions in India, including a complete smoking ban in public places, as well as a ban on tobacco advertising, promotion, and sponsorship [39]. The cost estimate assessed government costs for enforcing and monitoring the policy including strategic planning meetings, human resources, media and communication activities, and selected supplies and equipment costs [39]. Given the paucity of information regarding the cost of implementing and enforcing a complete smoking ban in a low- or middle-income country, the current analysis relied on model-based estimates [39].

The total annual cost of implementing and enforcing a complete smoking ban in combination with a ban on tobacco advertising, promotion, and sponsorship legislation in India is $0.08 per person in 2008 USD. The inclusion of the cost of implementing both policies is likely overestimating the cost to implement smoke free legislation on its own. The cost of implementing a complete smoking ban in Gujarat would be approximately $4,047,759 USD based on 2001 Census data and assuming a cost of $0.08 USD per person [22].

##### Cost of Implementing 2008 Rules (Partial Smoking Ban)

The cost of implementing and enforcing the current smoke free legislation as defined in the 2008 Rules was estimated for a one-year period from October 2008 when the Rules came into effect in the state of Gujarat. The cost analysis took into account central and state government expenditures for implementing and enforcing the current legislation, as well as the tobacco control infrastructure needed to accomplish these tasks. The specific programmatic costs included in the estimate included the following inputs: personnel time at state and district level for coordination, training workshops, as well as media and communication activities such as folk shows and print advertisements [38].

Using state tobacco control cell budgetary records, the state government spent an estimated $2,413 (2008 USD) per month and the central government an estimated $2,506 (2008 USD) per month to implement the current legislation over 12 months. The expenditure from the state government focused on implementation for the entire state of approximately 50 million people, whereas the central government funds were for two districts—Vadodara and Sabarkantha—covering 3 million people [38]. The total monthly expenditure by the state and central government was calculated per person with the assumption that the central government funds could be applied to all locations in addition to state funds for full implementation of the current legislation. The estimated total annual cost of implementing and enforcing the partial smoke free policy described in the current legislation in Gujarat is $59,036 USD in 2008 assuming a cost of $0.0012 USD per person [38].

### 2.4. Outcome Measures

Both cases of acute myocardial infarction (AMI) averted and life years (LYs) gained were used as outcome measures for this analysis. The number of cases averted was calculated to provide decision makers with a condition-specific outcome, whereas the outcome of LYs gained provides a summary measure for comparisons between health conditions in Gujarat. We chose LYs saved instead of Disability Adjusted Life Years (DALYs), which are typically used by international agencies, because LYs are easier for decision makers to understand, and thus are more applicable to policy decisions [48].

The anticipated reduction in SHS after implementation of a complete ban and a partial ban was used to estimate the reduction in AMI incidence based on a simulation model developed by Richiardi *et al.* 2009 [33]. This model calculated the expected drop in AMI incidence based on decreased exposure to SHS, as well as hours of exposure, and the relative risk of AMIs associated with exposure to SHS and active smoking. Using the expected reduction in exposure to secondhand smoke after a partial ban [27] and a complete ban [30], the current analysis relied on Richiardi *et al.*’s [33] calculated estimate of the anticipated reduction in AMI incidence, 0.017 and 0.05 for a complete and partial ban, respectively. This estimate represents only the effect due to the reduction in exposure to passive smoking after a complete and partial ban. The estimated risk reduction for the complete ban used in the current analysis was similar to the 17% pooled risk reduction in AMIs found at 12 months post-ban in two meta-analyses [34,35].

In order to estimate the LYs saved, we assumed a reduction in current smoking prevalence was attributed to quitting among current smokers. We assumed that smoke free legislation reduces the prevalence among current adult smokers immediately after implementation by motivating smokers to quit. In comparison, the change in initiation rate occurs more slowly due to shifting social norms over time as a result of the legislation’s implementation [49].

The total number of LYs saved by the 2008 Rules was estimated for an analytic horizon of ten years and discounted at 3%. Since there are no published studies, to our knowledge, on the number of LYs saved due to tobacco control interventions in lower middle-income countries, we based the estimates of life years gained after a smoker quits from a large prospective cohort study in the US [37]. This study began in 1982 and followed a cohort of 1.2 million US adults [37]. It calculated the years of life gained for smokers who quit at ages 35, 45, 55, and 65 compared to the life expectancies of those who continued to smoke [37].

The LYs gained for male and female smokers who quit were adjusted for India’s life expectancy. In India, life expectancy is 62 years for men and 64 years for women as compared to 75 for men and 80 for women in the US [50]. The adjusted LYs gained relative to continuing current smokers are presented in Table 2.

### 2.5. Sensitivity Analysis

Given the uncertainties in the precision of the parameters used in the cost-effectiveness analysis, we carried out sensitivity analyses. The range for each parameter was based on the available evidence. In the case of parameters where high and low estimates around the base case were not available, a standard convention of 50% more or less than the base case was employed. Best and worst case scenarios were estimated based on the high and low estimates of each parameter from the literature (Table 1).

## 3. Results and Discussion

Through implementation of a complete smoking ban as compared to the current partial ban contained in current COTPA legislation, 17,000 additional cases of AMI could be averted in Gujarat.

Based on the decision analytic model, implementing a complete smoking ban covering all public places is cost saving compared to the current COTPA legislation and 2008 Rules when the incremental cost-effectiveness ratio (ICER) is calculated with natural units, as well as with the summary measure of life years (LYs). When compared to the current partial ban, a complete smoking ban would save an additional 438,000 LYs based on the base case estimate that 3% of smokers would quit using cigarettes or bidis after implementation of the law. The results from the base case, as well as the best and worst cases compiled in the sensitivity analyses are presented in Table 3. The incremental cost-effectiveness ratios presented in Table 3 were calculated by first determining the net cost of the alternatives. The net cost was calculated by subtracting the medical treatment costs saved from the cost of implementing the legislation. Both the costs of treating AMIs, as well as a range of tobacco-attributable diseases, were used in the analysis. The ICERs were also calculated without accounting for medical treatment costs saved for comparison. A complete ban is highly cost-effective under each scenario considered in the analysis presented in Table 3. Furthermore, the base case and best case scenarios are cost saving. In the worst case scenario, it would only cost 262 USD (11,350 Rs) for each AMI averted and 56 USD (2,436 Rs) for each life year gained, well below the World Health Organization’s (WHO) threshold for cost-effectiveness, 880 USD (38,000 Rs) [51,52].

If medical treatment costs are not taken into account, the cost per LY and cost per AMI remain highly cost-effective in all three scenarios ranging from a cost of 2.24 to 112 USD (97 to 4,952 Rs) per LY gained and 37 to 387 USD (1,603 to 16,764 Rs) per AMI averted. The government of Gujarat would have a net savings of 36 million USD (1.6 billion Rs) if accounting savings from the treatment of heart disease and would save almost 1 billion USD (4.2 billion Rs) if all tobacco-attributable disease costs are considered.

Implementing a complete smoking ban covering all public places throughout Gujarat would be a cost saving alternative to the current partial ban described in COTPA and the 2008 *Prohibition of Smoking in Public Places Rules* for reducing tobacco-related disease outcomes. Our analysis demonstrates that implementing a complete ban in public places over the course of one year would reduce the number of AMI cases, and the life years lost associated with smoking-related disease, as well as save money for the government of Gujarat.

**Figure 1.**
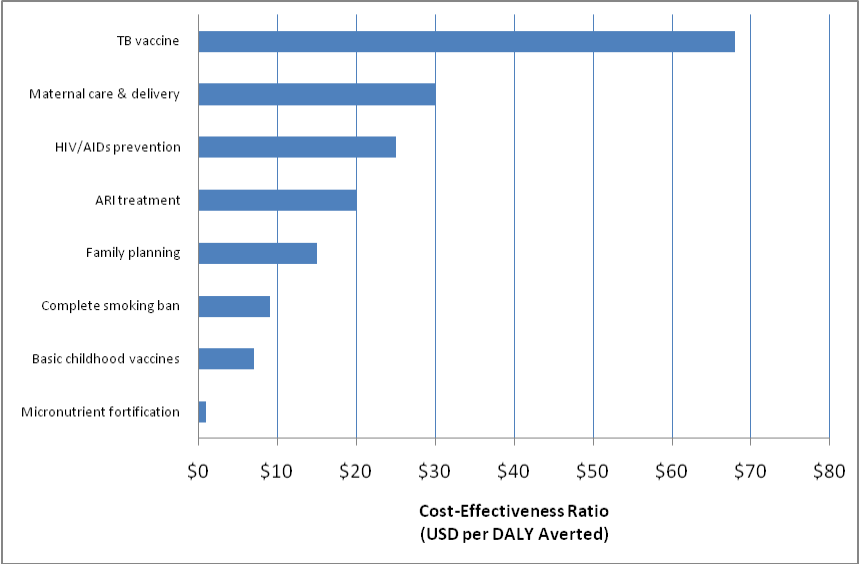
Cost per DALY averted for selected health interventions (adapted from [54]).

A 2006 regional cost-effectiveness analysis of the cost per disability-adjusted life year (DALY) averted in South East Asia for clean indoor air enforcement was 340 USD (14,719 Rs) per DALY averted [53]. The current study found implementation of a complete smoking ban to be similarly cost-effective, even without consideration of medical treatment costs saved and the years lived with disability that are included in the calculation of DALYs and may be averted by a complete smoking ban. In addition, when comparing the cost of implementing a complete smoking ban in Gujarat with other health interventions in low- and middle-income countries, as illustrated in Figure 1, it remains comparatively cost-effective [54].

Because the evidence on implementing smoke free legislation in India is lacking, it is unclear what the effectiveness of the complete ban on quit rates and reductions in secondhand smoke (SHS) exposure would be in Gujarat. Our analysis shows that if the complete ban is effective at reducing adult smoking prevalence by 0.12%, or reduces treated heart disease cases by 1% it would be cost saving. Furthermore, this level of effectiveness is likely a very modest estimate. A meta-analysis of the impact of smoking bans on AMI found an average reduction in AMI incidence of 17% [35]. Two longitudinal cohort studies in Scotland found a quit rate of 4% and 12% among bar workers and the general population, respectively, after implementation of a complete smoking ban [30,54].

### 

#### Limitations

Our analysis only compared the number of adult former smokers that would result from the two policy alternatives. It did not account for reductions in consumption among current adult smokers or reduced initiation of tobacco smoking among youth. In addition, the estimated impact of the two policy alternatives on AMI risk did not consider a reduction in current smokers; but rather, represented only the effect due to a reduction in exposure to passive smoking. As a result, this analysis likely underestimates the LYs saved and AMI cases averted, and a complete ban would be even more cost-effective [55].

Additionally, this study does not account for the loss of productivity from tobacco-related illness. These costs are likely to be considerable. A 2008 study of the direct medical costs of treatment of illnesses caused by exposure to secondhand smoke in the state of Minnesota in the US conservatively estimated these costs to be $228.7 million annually—or $44.58 per resident—with the majority of these costs related to two principal conditions, lung cancer, and coronary heart disease [56]. Including all of the costs to society from active smoking and exposure to SHS would further improve the cost-effectiveness of implementing a complete ban. Furthermore, the disease costs used in the analysis are conservative since the available data reflected the prevalence of treated diseases, not necessarily the prevalence of the disease. Therefore, the disease costs are very likely an underestimate of the cost of tobacco-related disease costs attributable to smoking and exposure to SHS in Gujarat.

In general, the current study determined the benefits of the policy alternatives using published literature or model-based estimates from international and regional analyses. The analysis relies on published estimates for the anticipated reduction in active smoking prevalence and secondhand smoke exposure in countries where the gender differences in tobacco use are not as substantial as the gender difference between male and female smoking in Gujarat. Therefore, this may result in the current analysis overestimating the number of female smokers expected to quit because of partial and complete smoking bans. However, the published estimates used for the effectiveness of partial and complete smoking bans were based on countries with largely similar gender profiles of tobacco use; and therefore, the incremental difference in effectiveness would not be significantly impacted.

Lastly, given the lack of available cost data on implementing a complete smoking ban in a low- or middle-income country, the analysis relied on a simulated estimate by WHO-CHOICE [39]. In addition, the cost estimate included both the cost of implementing a complete smoking ban, as well as a ban on tobacco advertising, promotion, and sponsorship [39]. Therefore, the analysis likely overestimates the cost to implement a complete smoking ban in Gujarat.

## 4. Conclusions

Despite these limitations, the strength of the analysis is that it maintains a conservative estimate and all scenarios illustrate that implementing a complete smoking ban in Gujarat would be beneficial. While this analysis was based on effectiveness evidence from high and middle-income countries, the cost analysis was conducted using national data from India and the findings are in line with regional estimates.

Given the prevalence of tobacco use and exposure to secondhand smoke in Gujarat, as well as the potentially considerable impact of smoke free public places, expanding COTPA’s Section 4 and the 2008 Rules to include all public places and increasing funding for enforcement through the National Tobacco Control Programme has the potential to deliver large health and economic benefits. The present study illustrates that implementing a complete smoking ban in all public places has the potential to produce significant health benefits and lead to economic savings for the government of Gujarat.

## Figures and Tables

**Table 1.** Parameter estimates and assumptions.

**Parameter**	**Base Case**	**Sensitivity Analyses Range**	**References**
**Epidemiologic Parameters**			
2001 State of Gujarat, India Population (>age 20)	50,671,017	n/a	[22]
Prevalence of Tobacco Smoking (age 15 and above)	0.198 males 0.015 females	n/a	[3]
**Intervention Parameters**			
Percent Change in Current Smoking Prevalence after Partial Ban^a^	0.015	0.01–0.02	[median]; [23,24]
Percent Change in Current Smoking Prevalence after Complete Ban^b^	0.0335	0.029–0.038	[median]; [25,26]
Percent Change in Exposure to SHS after Partial Ban^c^	0.22	0.21–0.27	[27–29]
Percent Change in Exposure to SHS after Complete Ban^d^	0.86	0.70–0.91	[30–32]
Percent Change in Hospital Admissions for Acute Myocardial Infarction after Partial Ban	0.05	0.01–0.10	[33]
Percent Change in Hospital Admissions for Acute Myocardial Infarction after Complete Ban	0.17	0.10–0.25	[33–36]
LYs Saved per Person that Quits Smoking	2.7	0.9–4.2	[37]
**Economic Parameters**			
Cost of Implementing Partial Ban	0.0012 per person (2008 USD)	0.0006–0.02	[38]
Cost of Implementing Complete Ban	0.08 per person (2008 USD)	0.04–0.12	[39]; [±50%]
Tobacco-related CHD Healthcare Costs per Person, incurred Annually for 10 years	2,291 (2008 USD)	124–4,459 (2008 USD)	[40]
All Tobacco-related Healthcare Costs per Person, incurred Annually for 10 years	615 (2008 USD)	50–4,459 (2008 USD)	[40]

a Base Case is the median between a partial ban in South Korea [23] and a simulated estimate based on US data [24];

b Base Case is the median between a complete ban in Ireland [25] and a pooled estimate of complete bans in the US, Australia, Canada, and Germany [26];

c Base Case is based on a partial ban in Spain [27] with high and low estimates from the Netherlands [29] and the US [28], respectively;

d Base Case is based on a complete ban in Scotland [30] with high and low estimates from Uruguay [32] and Norway [31], respectively.

**Table 2.** Adjusted LYs gained in India *.

	**Men**	**Women**
**SmokingBehavior**	**LYs Gained**	**LYs Gained**
Quit at age 35	4.2	3.6
Quit at age 45	3.4	3.3
Quit at age 55	2.1	2.5

* All discounted at 3%.

**Table 3.** Cost-effectiveness results.

	**Incremental Difference: Complete Ban*vs.*Current Legislation**	**Incremental Difference: Optimistic Case**	**Incremental Difference: Worst Case**
Gross Intervention Costs (C)	3,994,645	1,996,438	5,067,102
AMI Treatment Costs Saved (T)	40,051,602	237,927,598	1,626,740
All Tobacco-Attributable Treatment Costs Saved (T)	99,609,250	946,905,837	2,527,779
Net Costs (AMI Treatment) *(C-T)	–36,056,957	–235,931,160	3,440,362
Net Costs (All Tobacco-Attributable Disease Treatment) * (C-T)	–95,614,605	–944,909,399	2,539,322
AMI Cases Averted (A)	17,478	53,361	13,109
Smokers Quitting (Q)	221,154	385,100	46,060
Life Years Gained (L)	437,589	891,945	45,268
**Incremental Cost-Effectiveness Ratios**			
Cost per LY Gained (C-T)/(L)	***Cost Saving***	***Cost Saving***	*56 (USD) Highly Cost-Effective*
Cost per LY Gained w/out Medical Treatment Saved (C/L)	9.13 (USD) *Highly Cost-Effective*	2.24(USD) *Highly Cost-Effective*	112 (USD) *Highly Cost-Effective*
Cost per AMI Case Averted (C-T)/A	***Cost Saving***	***Cost Saving***	*262 (USD) Highly Cost-Effective*
Cost per AMI Case Averted w/out Medical Treatment Saved (C/A)	229 (USD) *Highly Cost-Effective*	37 (USD) *Highly Cost-Effective*	*387 (USD) Highly Cost-Effective*

* The net costs were calculated by subtracting the medical treatment costs saved from the gross cost of the intervention.
